# Coping mechanisms and strategies adopted to improve the quality and timeliness of immunization data among lower-level private-for-profit service providers in Kampala Capital City, Uganda

**DOI:** 10.1371/journal.pone.0303998

**Published:** 2024-08-28

**Authors:** Eric Ssegujja, Martha Akulume, Elizabeth Ekirapa-Kiracho, Paul Kiggundu, Sarah Karen Zalwango, Elizeus Rutebemberwa

**Affiliations:** 1 Department of Health Policy Planning and Management, School of Public Health, College of Health Sciences, Makerere University, Kampala, Uganda; 2 Directorate of Public Health and Environment, Kampala Capital City Authority, Kampala, Uganda; Freelance Consultant, Myanmar, MYANMAR

## Abstract

**Background:**

Lower-level urban private-for-profit health service providers are actively engaged in the delivery of immunization services. However, not much is known about their everyday endeavours to improve data quality and ensure the submitted data meets the quality and timeliness requirements as per established guidelines. The objective of this paper was to examine the coping mechanisms and strategies adopted to improve the quality and timeliness of immunization data among lower-level private-for-profit service providers in Kampala Capital City, Uganda.

**Methods:**

A qualitative study design was adopted with in-depth interviews (n = 17) and key informant interviews (n = 8) completed among frontline health workers, district health managers and immunization implementing partners. Analysis followed a thematic approach with coding conducted using Atlas. ti, a qualitative data management software.

**Results:**

Overall, coping mechanisms and strategies adopted to improve the data quality among lower-level urban private-for-profit immunization service providers included; Experiential attachment for practical skills acquisition in data management, data quality peer-to-peer learning among private-for-profit immunization service providers, registration using cohort system for easy tracking of records during subsequent visits, separation of visiting service user records from regular attendants, service delivery modifications such as reward for better performance, engagement of Village Health Teams (VHTs) in outreaches and data completion, and data quality checks through review of monitoring charts to identify gaps in data quality.

**Conclusions:**

Within the urban context, the delivery of immunization services by lower-level private-for-profit services faces data quality challenges some of which stem from the implementation context. Different coping strategies focusing on frontline health worker skills, enhanced experience sharing through peer-to-peer learning, modifications to registration and routine review of monitoring charts. However, these efforts were often faced with chronic barriers such as the high staff turnover calling for dedicated efforts to optimize the available implementation opportunities like guidelines mandating the public health facilities to supervise the lower-level private immunization service providers within their catchment areas to respond to the identified gaps.

## Background

Globally, immunization services are facing challenges to improve data quality as available data may not be able to be sufficiently used to improve service coverage for optimal performance [1]. The suboptimal quality of immunization data is a troubling and major problem among immunization program managers and implementers [2]. Concerns about the quality of immunization data are increasingly growing to crisis levels [3]. Without interventions to mitigate this challenge, data quality often deteriorates and compromises immunization service coverage through the absence of evidence-based planning [3]. Currently, efforts to improve immunization coverage are increasingly turning to data quality improvements to inform evidence-based interventions [4, 5].

Given the importance of data in informing planning and decision-making for immunization services, it provides a case for an investigation into current data management practices among the lower-level urban private-for-profit immunization service providers [4, 5]. Compromised immunization data quality is a common problem within health systems, especially among private service providers [2]. This is partly due to the loose supervision extended to such service providers compared to their public sector peers [6]. Other factors relate to the inability to recruit and retain data management personnel to streamline data systems [7].

Within the urban immunization service delivery context, there are several examples citing cases of failure to use available data to improve immunization coverage [8]. Currently, the rural-urban migration wave is forcing several younger people in their reproductive age groups into cities in anticipation of better economic opportunities [9]. The transactional urban lifestyle makes urban dwellers highly mobile and busy during the day rendering the available immunization services offered during the day inaccessible to many which extends to data quality [10]. When considering the complexities surrounding the quality improvement of immunization data, it is important to factor in the urban context [11]. Immunization services are predominantly delivered through the private sector in urban areas while current efforts to support the improvement of immunization data quality largely focus on public and private-not-for-profit facilities [12, 13]. This drives improvement efforts towards technical rather than contextual solutions.

The adopted health worker practices in a self-improvement bid have the potential to offer lessons for intervention designs aiming at improving immunization data quality. Health workers unable to record and submit quality and timely immunization data develop coping mechanisms that enable them to cover up the existing gaps and to collect and submit available data [14, 15]. Eventually, the quality gets compromised and initially, the data gaps may only be unveiled when vaccine supply does not tally with service utilisation or later when vaccine-preventable diseases resurgence is observed. To meaningfully engage the private sector in immunization data improvement, there is a need to increase our understanding of their perspectives regarding data quality and efforts undertaken to improve it. This paper aims to examine the coping mechanisms and strategies adopted to improve the quality and timeliness of immunization service data among lower-level private-for-profit service providers in Kampala capital city of Uganda. By lower level, we refer to the first levels of clinical service provision within the tiered health service delivery structure. These are categorised as Health Centre Two (HCII) as the first tier and Health Centre Three (HCIII) as the second tier of clinical health service provision. Among the services provided, immunization is among. The same structure is followed for both the public health facilities and the private-for-profit providers where licensing of the same follows these structures. In this case, we shall use the lower level to refer to the private-for-profit immunization service providers at those two levels of service provision.

## Methods

### Study design and setting

The study, part of the research program aimed at improving immunization data quality among urban lower-level private immunization service providers utilised a cross-sectional qualitative design and sought to capture the coping mechanism and strategies adopted to improve data quality. The study aimed to identify coping mechanisms and strategies adopted to improve the quality and timeliness of immunisation data among lower-level private-for-profit service providers in Kampala capital city, of Uganda. Kampala is the capital of Uganda and the major commercial centre in the country. It has a resident population of 1.6 million but this number surges during the day to approximately 4 million people, especially in the central business district. Since 2011, the city has been administered by the Kampala Capital City Authority (KCCA), a corporate structure overseeing the five geographical administrative divisions with several specialist directorates among which is the Directorate of Public Health and Environment responsible for planning and delivery of the city’s health services.

In terms of health service delivery, the city has characteristics of a pluralistic health system with a tertiary-level public sector presence where all the national referral hospitals are based within the city. The private not-for-profit is dominated by faith-based health facilities managed by the respective medical bureaus. The city has a strong presence of private-for-profit service providers at all levels ranging from the high-level corporate hospitals offering diagnostic and specialised services to lower-level clinics offering mainly maternal and child health services of which immunization forms part of the continuum. About 98% of the health service provision in the city is through the private sector with a total of 1,497 health facilities providing care to an estimated 4-million-day population. 94% of the facilities are private-for-profit, 4% Non-Governmental Organisation (NGO) owned and 2% government-owned with most (93%) at level II health centres, the lowest level of clinical service provision [16]. Details of health facility ownership are provided in Table 1 below.

**Table 1:** Health facilities in Kampala by the level of operation and ownership.

Facility level	Ownership				% by level
	Govt	NGO	PFP	Total	
National Referral Hospital	4	0	0	4	0.27%
Hospital	3	6	14	23	1.54%
HCIV	1	2	8	11	0.73%
HCIII	6	17	30	53	3.54%
HCII	12	36	1,358	1,406	93.92%
Total	26	61	1,410	1,497	100%
% by ownership	2%	4%	94%	100%	

Source: KCCA Directorate of Public Health and Environment annual report 2019/20

Immunization services within the city are delivered through the public sector, private-not-for-profit and private-for-profit health facilities. These follow the recommended immunization schedules from the Ministry of Health’s Expanded Program on Immunization (EPI). Recently, other vaccines were added to the national schedule such as Pneumococcal Conjugate Vaccine (PCV), Human Papilloma Vaccine (HPV) and Rota which are delivered within the city through similar arrangements. Whereas the public and private-not-for-profit facilities are supported through Primary Health Care (PHC) funds to deliver immunization services, the private-for-profit is only minimally supported through the provision of the vaccine, cold chain support, data tools and support supervision to deliver the same. They are in turn expected to submit immunization utilisation data to KCCA in return for receipt of subsequent vaccines free of charge which they are expected to also offer free of charge to their clients. It is within this context that efforts to improve the quality of immunization data and the steps taken to achieve it occur.

### Study population

**Fig 1:**
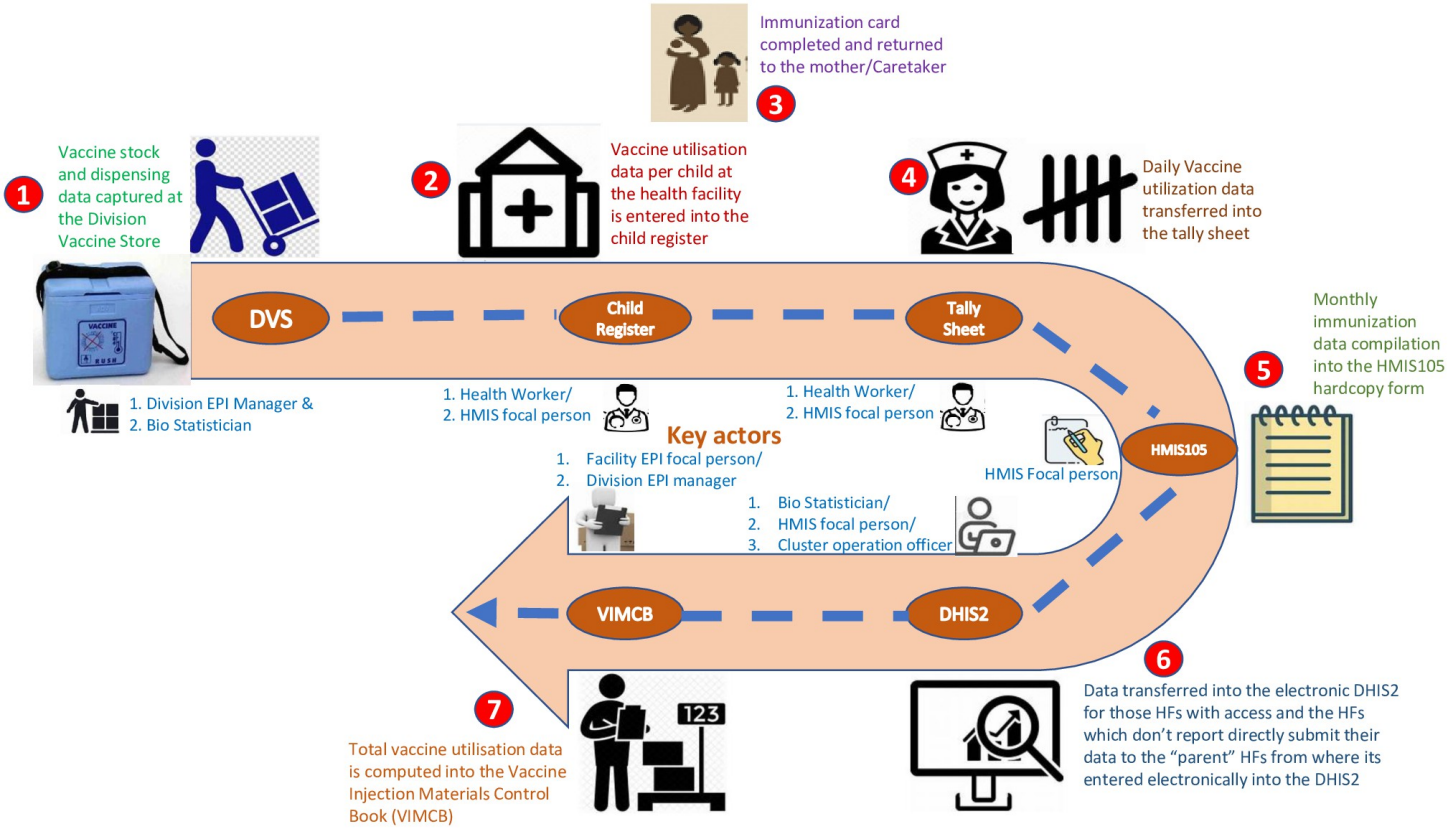
Immunization data hierarchical structure.

The study population primarily comprised frontline health workers delivering immunization services from the private sector and are responsible for recording the utilization data, health facility managers from both the private and public health facilities within the city, division and city health managers who are responsible for ensuring that data systems are functional according to the immunization data hierarchical structure (Fig 1), and immunization services implementing partners and other key stakeholders that support immunization services and also data systems within the city.

### Sample size and sampling procedure

A purposive sample of twenty-five respondents was recruited for this study. The inclusion criteria primarily focussed on the individual’s contribution to the delivery of immunization services within the city. On the one hand, health facilities from where respondents were recruited represented the different levels of health service provision starting from the lowest level offering clinical services (HCII) up to the Hospital level where some reported their immunization data and it happens to be the highest level among private providers. These. On the other hand, the health managers also represented the different levels of health systems management which included facility management, division health services management and Capital City Authority health services management. We recruited until saturation was obtained with the final sample including frontline health workers (n = 17) who were drawn from the two city divisions which included Makindye and Kawempe Division, division EPI personnel (n = 4), city health managers (n = 3) and Immunization implementation partners (n = 1). Participants were eligible when they expressed willingness to take part in the study, were working directly with the delivery of immunization services, were familiar with immunization service delivery or were managers overseeing the delivery of immunization services. Details of study participants are presented in Table 2 below.

**Table 2:** Characteristics of study participants.

	Characteristics	N (100%)
**1**	**Age**	
	20–30 years	9 (36%)
	31–40 years	8(32%)
	41–50 years	6(24%)
	>51 years	2(8%)
**2**	**Gender**	
	Male	7(28%)
	Female	18(72%)
**3**	**Cadreship**	
	Data personnel	3(12%)
	Nurse	7(28%)
	Midwife	11(44%)
	Clinical Officer/Doctor	4(16%)
**4**	**Duration spent in practice**	
	< 5 years	13(52%)
	6–15 years	10(40%)
	>16 years	2(8%)
**5**	**Years spent at current job**	
	<3 years	15(60%)
	4–10 years	8(32%)
	>11 years	2(8%)
**6**	**Role in immunization services**	
	Data entrant only	3 (12%)
	Vaccinator/ data entry	18(72%)
	Health manager	4(16%)
**7**	**Respondent category**	
	Frontline health workers	17 (68%)
	EPI focal persons	4 (16%)
	Health managers	4 (16%)

### Data collection

Data collection was conducted between June and July 2021 by the first author together with three graduate-level research assistants one of whom was the female who doubled as a research officer on the project. Potential respondents were approached at their places of work where the aims, objectives and procedure of the study were explained. For those who expressed willingness to participate in the study, face-to-face interviews were organised. A secure and private place was organised from where the interviews were conducted. All participants gave written informed consent before administering the interview. A digital audio recorder was used to record all sessions while the interviewer took down notes. The data collection tool developed by the study team specifically for this study was used to guide the flow of the discussions. Specifically, the data collection tool explored; 1) the current status of immunization service provision, 2) immunization data reporting arrangements, 3) data quality issues, 4) challenges experienced by private-for-profit service providers, 5) coping mechanisms, and 6) Strategies adopted to address the challenges. The data collection tool was pre-tested at two health facilities other than those that took part in the study with feedback incorporated to improve the clarity of the questions. Overall, interviews lasted between thirty minutes to one hour. All interviews were conducted in English and thereafter transcribed verbatim into a Microsoft Word document.

### Data management and analyses

Transforming health workers’ immunization data management experiences into the textual expression of meaning was guided by a reflexive approach adopting themes and subthemes as structures of meaning. This involved the following steps; 1) data familiarisation through reading the narrative display within a sample of five transcripts, 2) development of the codebook which was discussed with variations resolved among the team, and 3) entering all transcripts into Atlas. ti a qualitative data management software, 4) inductive and deductive coding of all transcripts considering line-by-line text and sentences where textual data relating to the code were highlighted from the transcripts and attached to the corresponding code, 5) a manual pile sorting and abstraction of coded data into themes and sub-themes which involved amalgamation of data clusters following hermeneutic circles, and lastly, 6) Interpretation, triangulation, methodological synthesis and write-up of results which have been organised into broader themes. The Consolidated Criteria for Reporting Qualitative Results (COREQ) checklist was used to guide the reporting of results where typical quotes have been used to reflect respondents’ voices. Triangulation was done by comparing results from the different data sources while pseudonyms have been used for direct quotes.

#### Ethical approval and consent to participate

This study received ethical approval from Makerere University School of Public Health Higher Degrees Research and Ethics Committee (HDREC) **ref; HDREC 848**. The final ethical clearance was processed by the Ugandan National Council for Sciences and Technology (UNCST) **ref; HS996ES.** Further administrative clearance was obtained from the management of all participating health facilities and organisations where the study was conducted. All participants gave written informed consent for study participation.

## Results

In this section, we present coping mechanisms and strategies adopted by the lower-level private-for-profit health facilities to improve the quality and timeliness of immunization data in an urban setting of Kampala City in Uganda. These strategies are organised around the major theme which includes; 1) Health workers’ data management skills improvement, 2) innovations around registration and data systems, 3) service delivery modifications, 4) implementation of data quality checks and 5) performance reviews conducted during mentorship visits.

### Health worker data management skills improvements

The strategies that were implemented targeting improvement of data handling skills among the health workers included; 1) experiential placement of private health facility workers at public facilities to acquire practical skills in handling immunization data, and 2) peer-to-peer learning among private health workers through their division platforms. It was observed that initiatives to refresh health worker skills saw health workers from the private facilities matched through experiential attachment to public facilities to learn how immunization data was managed. For the case of recruits, they took advantage of training organised by the division Expanded Program on Immunization (EPI) focal persons to send their health workers to attain the required skills while current staff requiring additional skills, were mentored in data quality improvement through onsite support to translate those skills into timely submission of quality data.

*For those who are starting, we attach them to a public facility here when they express interest to begin conducting immunization services which they have to put in writing to the Division Medical Officer (DMO). We are then sent to assess the facility’s capacity so that we can either qualify it or not to begin immunizing. So, we have to attach them to a public facility for a two-week hands-on training in immunization activities starting from the vaccination point up to records, documentation, and cold chain then we can recommend them to start offering the service.*

***(Health manager_02)***

Peer-to-peer learning and support efforts were undertaken toward immunization data quality improvement among health workers. Within particular city divisions, health workers from private immunization service providers formed groups from where they would communicate experiences and challenges related to immunization data management. Peers would then respond with suggestions on how they responded to the same when they were faced with similar or related challenges at their particular places of work. These interactions would then form how individual health workers adapted or adjusted to suit their particular work context and data requirements. Learning from peers enabled health workers to adapt the same to their context to improve timeliness and submission of quality data.

Lastly, instances, where radical approaches to cajole private providers to improve quality data, were also reported. Particularly it emerged that when private facilities were slow to improve, a name-and-shame approach was used.

*The only penalty we have is shaming, because for quarterly review meetings, we list the good-performing and the poor performing, we just name and shame and end there*

***(Health manager_01)***

### Innovations around the registration systems

Regarding innovations around data and registration systems, the strategies adopted included; 1) using the birth cohort system, 2) recording clients’ telephone contacts for easy follow-up, 3) using the appointment book to track defaulters, 4) separating visitors from regular clients, 5) introduction of unique client identification numbers and 6) improvising through photocopying of immunization data tools to mitigate impending stock-outs. Registration following birth cohorts to improve immunization data quality among private providers was adopted to address barriers arising from the registration system currently in use. Private-for-profit providers documented vaccination records in the register according to the birth month of the child to facilitate easy tracking of child immunization records during subsequent visits. Linking new entries to previous records helped them respond to the data completeness challenge which had persisted thereby boosting the quality of immunization data.

*Ok, we thought of two strategies, one, maybe it would be much easier if we register these children in birth cohorts the way they do for HIV. Let us say children born in January alone are in their book and if I want to get the information about a child, I just look at the dates of birth of the child then I know which book to find the child.*

***(Health worker_01)***

Some private providers designed the record systems to ensure they captured the telephone contacts of all service users. When records were reviewed and an observation was made that a certain service user had missing immunization data due to an incomplete schedule, providers would consult the same records to obtain their telephone contacts, reach out to them to either bring the child back for immunization and have that record updated or find out if they had completed the vaccination from elsewhere and reconcile the records. Relatedly, other private facilities maintained an appointment book. This extended to updating details of the subsequent appointment dates. They would then use this information to remind the caretakers not to miss their next appointment which saw a reduction in missed appointments and data incompleteness.

Recording visitors and regular clients in separate registration books was deemed necessary by some private immunization service providers to address the high dropout rates occasioned by the transient urban population who sought immunization services from multiple service providers in the city. In pursuit of the same objective, other private providers would record them at the back of the immunization register in order not to mix them with regular resident service users which made records retrieval difficult hence compromising data quality in some stances. This enabled health workers to easily locate and link records of visitors and regular attends upon return to minimise unlinked data cases which improved data completeness.

*What we do, is we have a visitors’ book in the child register. It is designed in a way that when someone has missed a dose or someone has come from a different facility, we register them there. The [client] crossing [from one facility to another] is there in different facilities*.

***(Health worker_07)***

Private immunization service providers considered issuing clients unique identification numbers for easy tracking. In other instances, appointment books were introduced which would guide private providers on those who had not honoured the appointment and inform their follow-up of defaulting mothers to address the missing data and improve data completion rates. This process improved the easy linking of immunisation data to address the challenge of data incompleteness.

*So, we have an appointment book which we use for general appointments for all services. So, we just place the child’s number, the mother’s number and the child’s name, so we call them to remind them about the next due date for immunization that’s what we always do and try to emphasize that please bring your baby back on this day for immunization. Anyone responsible for the appointment book follows up and then the person who is in charge of immunization goes back again and tries to look through and see those who were supposed to come back but possibly missed*

***(Health worker_04)***

Private immunization service providers also considered making photocopies of the tools if the provided immunization data capture tools were deemed insufficient. This would address the shortfall hence minimizing the loss of data in the absence of the tools to record.

*there [are times] when some of them get finished, so we have to wait but what we do we photocopy, then we can carry it on. For example, if I may tell you we have the monitoring reports whereby we need them in place so you find that maybe we have to photocopy*

***(HMIS focal person_02)***

### Service delivery modifications

Private immunization service providers also highlighted that among the strategies adopted to improve data quality and timeliness which hinged on service delivery modifications were; 1) the introduction of a reward system for mothers who comply with the recommended immunization schedule to enhance high completion rates, 2) task shifting of data entry to VHTs when clinical workers were overwhelmed with the workload, 3) institution of data completion outreaches in the facility’s catchment areas. Specifically, good-performing clients would be appreciated and rewarded for completing of immunization schedule on time to address default rates which would lead to incomplete and missing data when not attended to. Rewards were in the form of the distribution of a mosquito net and sometimes such mothers would be appreciated and offered an opportunity as role models to speak to other mothers during health talks about the value of timely vaccination of children. Such efforts encouraged other mothers to also make efforts to complete the immunization schedules for their children which addressed data completeness.

*we were giving some rewards to a mother who had completed the schedule we gave some rewards like the [mosquito] nets as a sign of appreciation and even gave a slot to talk to other mothers and how good it is to complete the schedule*.

***(Health worker_02)***

Private providers also highlighted that task shifting of record taking to VHTs, freed especially health workers with a heavy workload which enabled them to concentrate on providing immunization services while the VHTs performed the recording. It responded to the inherent challenges of fewer health workers amidst heavy client load typical in high volume private facilities would sometimes record inaccurately or forget to record some cases into the register. The VHTs working alongside private immunization service providers were also reported to engage in the registration of all children in the health facility catchment area which facilitated the computation of the target population that was used to estimate the denominator used to ensure accuracy of immunization data. Besides, the VHTs were engaged in mobilization and tracking down defaulters to address the missing data. With support from the immunization implementing partners they supported data capture both at the facility and in the catchment, area as revealed in the quote;

*There is a community health worker who works with the midwife and that is the one who helps in filling [out] the tools. The process of weighing the child, taking records and immunizing is too much for one person which is why they help. There always have to be two or three people helping there. The technical part is always done by the midwife like giving out the vaccines*

***(Health worker_09)***

Immunization data completion outreaches were another strategy to enhance the quality which focused on particular health facility catchment areas. Because private immunization service providers kept contact with their service users and given that urban catchment areas are relatively small geographically, private providers engaged in outreaches specifically for mothers who would not be reached via telephone contacts. Sometimes they would adjust their working hours to extend into the late evening to cater for those who had limited time during the day. In addition, private providers opened for immunization service delivery during weekends to extend services to urban dwellers who could not find time during the week to improve immunization schedule completeness which also translated into minimising opportunities for incomplete and missing data.

### Data quality improvement checks

City health systems managers and development partners in a collaborative partnership aimed at improving immunization services within the city supported private immunization service providers to which they also reciprocated by conducting data quality background checks. The strategies adopted prioritized; 1) reviewing monitoring charts at the facility level, 2) data quality checks within the DHIS2 by city health data managers, and 3) tailoring support to respond to gaps identified during data quality checks. At the health facility level, the use of monitoring charts to track performance was particularly highlighted during the supervision visits and micro-planning process. This facilitated the identification of data gaps at the facility to which health workers responded by addressing the underlying cause of such gaps.

*The quality of immunization first of all, as I said when you have a micro plan, you can identify the gaps. We use the monitoring charts for identifying the dropout rates and also since we all know the target population, we can evaluate ourselves at the end of the month.*

***(HMIS focal person_02)***

Continuous data quality checks performed by the KCCA health data team to ensure compliance with and performance of the divisions and specific private health facilities were deemed necessary in steps to improve data quality. Following the submission of data through the DHIS2, background spot checks on the submitted data were performed. This prompted data validation visits to particular private health facilities to physically verify and mentor concerned personnel on how to address the issue that may have caused data discrepancies. In particular, hard copy versions would be pulled out to make a comparison with what had been submitted through the DHIS2 system. Any discrepancies identified would be addressed and teams oriented on how to address such to avoid future recurrence of the same.

*they have a support supervision team that comes to check on the fridges, comes to check on the data, come to do the data validation, so they check and they will tell you I did a Data Quality Assurance (DQA) and everything is ok or I did and this is where you need to improve what’s what they always do there is a support team*

***(Health worker_08)***

Knowledge regarding challenges compromising data quality enabled city data supervisors to tailor capacity building that responded to the skills gaps identified. These were addressed during the subsequent supervision visits to those private health facilities. Generic content was developed in cases where the gaps identified were cross-cutting. These took different forms with the common ones including; one-to-one mentorships for individuals, particularly those health workers who handled data, and feedback meetings which involved several health workers within the private health facilities.

*there is a team, that’s DIT [data improvement team] we always move to these facilities for support supervision on immunization data. We go through these facilities one by one and look at their books, and then the data submitted and we can observe and compare. That’s when you are even able to know why then when you try to dig out what could have been the cause*

(Data manager_02)

the only thing we have been doing is to continue going for support supervision and mentorships. The other thing we do is give feedback and review meetings, we give feedback or even try to call.

***(Health systems manager_01)***

### Performance reviews

Performance review meetings conducted at the private health facilities were aimed at facilitating immunization data quality improvements. Participants reported that these performance review meetings were organized particularly for the health facilities which were deemed poor performers to share their performance and provide feedback to individual health facilities informed by identified gaps within the submitted data. This would allow all facility data handlers to go through a case-by-case review of all the problematic data gaps and thereafter have each addressed.

*those who are having challenges are called and we do performance reviews, people are asked if your facility is not performing well so they also feel bad when you tell them the facility is not performing well among all the other facilities so they go back and try to improve on that*

***(Supervising Health worker_03)***

### Mentorship

Lastly, owing to the high staff turnover in private facilities, facility managers and their proprietors worked out mechanisms of mentoring new health workers in data capture skills through experiential attachment at public health facilities. This opportunity was worked out through the city and division EPI department and the host public facility that facilitated its smooth implementation for all private immunization service providers who expressed a wish to benefit from it. It responded to the tedious process of having to repeat the same orientation and training that was offered to new health workers by the private health providers whenever there was a staff exit. After the experiential learning and mentorship at the public health facility, the new health workers then returned to the private facility to offer the services with the expectation that they would translate what was learnt during the mentorship into improving their health facility immunisation data.

*We have created groups for one-on-one like [within the] central division, we have a group for the EPI focal persons from those [private health] facilities from where we share and anybody who has a challenge in reporting so they send them and we can discuss it. Another way is when we move for support supervision we get one-on-one we see where the person has a challenge then we can discuss and see how we can improve on it.*

***(Health facility manager_05)***

## Discussion

In this study, the results suggest that lower-level private-for-profit immunization service providers in Kampala’s capital city adopted coping mechanisms and strategies to improve the quality and timeliness of immunization data. We report on coping strategies adopted by lower-level urban private-for-profit immunization service providers in data quality improvement efforts through timely and accurate reporting.

### Data quality management skills

Private immunization service providers enhanced their immunization data quality by engaging their health workers in experiential attachment to public health facilities for data quality mentorship placements. Within the urban immunization service delivery context, this was deemed appropriate because, unlike the private providers, public health facilities experience relative stability of their health workers including data personnel. This is mainly attributed to the security of tenure which enables such staff to grasp most of the immunization data requirements hence the ability to share lessons with fellow health workers from private providers who may be lacking in such skills. Besides, public health facilities hold the mandate to supervise and support lower-level private-for-profit immunization service providers within their catchment areas. It is therefore easier to extend that support to include capacity building in immunization data quality through experiential attachment. The other aspect is the requirement for all lower-level private-for-profit immunization service providers that do not submit their facility data directly through the DHIS2 system to submit their HMIS data through the supervising public health facility. To ensure quality, therefore, private health facilities go to the extent of taking their health workers for practical attachment at the public health facilities for experiential learning. They acquire practical knowledge on data aspects they need to focus on to have their data submitted on time and in good quality. As seen elsewhere, building capacities of health workers in data quality aspects is key for any immunization data quality improvement interventions [1, 17]. It is even boosted when interactive approaches such as coaching are implemented [18].

Results also revealed that among the strategies to boost the data management skills of health workers from the private immunization service providers was the data quality peer-to-peer learning that was undertaken. They would meet physically during organised meetings but would also attend to pending issues via online platforms. This aligned well with the urban immunization service delivery context whereby the private providers at different levels of capacity in terms of data management. Some had advanced data systems which were already digital while others were still using paper-based systems. Besides, the rates of staff turnover among these private providers tended to be high and it affected the in-house data management capacities of private providers differently. Other than seeking capacity building from external sources, it was rather appropriate that available capacities were shared among health workers from different private immunization service providers through peer-to-peer learning sessions. Engagements with networks of health service providers have been observed to improve vaccine coverage including data quality but also in ensuring full facility engagement [18]. Besides, the utilisation of digital platforms such as short message systems (SMS) or encrypted message platforms has been noted to improve routine immunization data quality [19].

### Innovations around data registration systems innovations

Some challenges impeding immunization data quality as our study revealed emanated from the data systems used to collect and submit that data. The existing data systems were designed to enhance uniformity and standardisation for all immunization data across sites. Whereas this was the case, the urban context dictated otherwise. Results revealed that private providers opted to register immunization data using the cohort system which followed registration by birth month. It was deemed a solution to challenges associated with record tracking when children returned for subsequent doses. Within the private health care setting, they also found it appropriate to use appointment books even for immunization services. This would offer them an opportunity to know the numbers to expect on a particular day and the doses they were coming for. In case those particular children did not turn up, they would then follow them up to ensure they returned for and completed their doses to minimise the missing data. It appears that these modifications were introduced to ease the workload but also ensure smooth flow and lessen the work while tracking immunization records. In supporting private immunization service providers to improve their data quality, it is therefore important to tailor such efforts to a context which lessens the work and does not introduce a heavy workload. As reported elsewhere, increasing the efficiency of health workers through addressing time-consuming data-entry processes is key to addressing immunization data-related challenges [20].

It also emerged that private providers adopted the registration of children while separating the visitors from regular attendance at their health facilities. This was done to facilitate tracking completion, especially for those visitors who sought different doses from different private immunization service providers. Further, some private providers opted to use unique client identification numbers. This practice is not new among private health service providers while delivering other health services as it facilitates linkage of facility attendance, and medication prescription while linking cost to services received. But within the context of immunization services where private providers are offered free vaccines, cold chain and data tools by the EPI in anticipation that they will provide the vaccination services free of charge to the users, it was a unique way of ensuring data quality. Unique client identification can facilitate easy tracking of defaulters and can be used to analyse data completeness trends over time within a facility and how that compares with other health services offered at the same facility. It also links to other subsequent health services sought by that same child from the same private health facility. Opportunities to link immunization data improvement with other health priorities were highlighted as important in ensuring quality and effective implementation [17].

### Service delivery modifications

From this study, we also learnt that private immunization service providers adopted strategies which modified their health service delivery in a bid to improve immunization data quality and minimise chances of missing data. Within their local arrangements, some private providers introduced a reward system for mothers who completed their children’s vaccination schedules on time. They would give parents/guardians appreciation gift hampers on behalf of their children related to their health and well-being such as treated mosquito nets. Others would be recognised and offered a platform to speak to fellow mothers seeking care at the private health facility about their children’s health and the value of completing their vaccination schedule on time. This is an important motivation to mothers but it also played well in helping the health facility have mothers who seek immunization services from them ensure they completed all mandatory vaccines which minimised missing and incomplete data. The gesture of reward with gifts such as mosquito nets comes with added values of increased ownership of benefits accruing from the child vaccine protection but also increased use of mosquito nets to prevent malaria in children [21]. Besides, it also improves the client-health worker relationship breeding improved communication that can eventually lead to an early notification to the health worker by the mother in case the child develops any health complications in future. In terms of immunization data improvement, the ability of the mother to talk to fellow mothers boosts their confidence but can also serve to influence other mothers to complete their children’s vaccination schedules on time to reduce default rates but also address incomplete immunization data.

Relatedly, the study results revealed that task shifting some of the data recording roles to the Village Health Teams (VHTs)-a community health worker cadres in Uganda-by some private immunization providers enhanced their data quality improvement efforts, especially for those data roles that were not so technical and fitted well the VHTs’ level of skills. Other private providers opted to have immunization and data completion outreaches where they still engaged VHTs to support this activity. They would organise outreaches to their catchment areas, have the VHTs check on the children and ask the mothers whether they had completed the vaccination schedules of their children. Whereas this was done to address the default rates, it also helped in addressing missing data, especially for those children who had sought subsequent doses from other private health facilities and did not report back to the previous service provider about completing the schedules from elsewhere. This action was particularly important as it responded to the contextual challenge of urban community health services whose service delivery is predominantly through the lower-level private-for-profit health service providers. Whereas there are a few public facilities where VHTs would ordinarily be attached, they tended to support the same function of tracking down unvaccinated children through working with private-for-profit health service providers which is not the same case for rural private health service providers. Such innovation helped in addressing the immunization default rates and missing data. Within the context of limited human resources, task shifting has been reported to address the human resource gaps especially when non-clinical/specialised roles are delegated to lower-level cadres [22]. In our case, recording immunization data at the facility with good guidance would effectively be done by these health worker cadres.

### Data quality checks

Some of the other strategies adopted by the private immunization services providers to improve the quality and timeliness of data were the review of monitoring charts to establish the performance which also informed them about the quality of data they were using to conduct these reviews. Auditing immunization data has been associated with the identification of data gaps as well as guiding targeted measures to respond to identified gaps [23]. For our case, it also extended into the data quality checks within the DHIS2 whose end action was to tailor support based on the identified data quality gaps. This was particularly important for the urban context where private-for-profit immunization service providers who form the bulk of the lower-level immunization service provision do not submit their data directly into the DHIS2 but rather first submit it to the public facility which later forwards it into the DHIS2 system. Such procedures may lead to compromise in data quality and can only be rectified through regular quality checks within the DHIS2 which are then reconciled with physical supervision visits at each of the private-for-profit immunization service providers. By instituting quality data checks, it would help unveil recording errors, incomplete indicator level data and implausible dates among others. Remaining intentional about the immunization data completeness, timeliness, and integrity among others has been reported as key in ensuring its quality and usability [4]. As reported elsewhere, this can be enhanced through timely feedback about data quality to the data collection teams during interactive sessions such as data review meetings [1, 7, 24].

One of the key aspects of complex adaptive systems is the ability for self-learning which breeds ongoing quality improvement efforts for better service delivery [25–27]. It emerged from this study that there were ongoing improvement efforts among private-for-profit immunization service providers that were either self-initiated or responded to particular challenges identified by the data personnel from the KCCA Directorate of Public Health. Whereas some were initiated at the health facility level, others originated from the oversight observations by the supervision teams. Performance monitoring through review meetings was one such effort that informed improvement initiatives targeting areas of weakness. These usually took the form of inviting all health workers involved in the delivery of immunization services to attend and provide feedback. After submission, background checks would be made on the data to establish its accuracy. These were also common coping strategies for data quality improvement among private-for-profit providers. Such factors highlight the importance of supporting private-for-profit providers in addressing data challenges starting from their ongoing efforts towards the same. It is paramount to build on such initiatives because they were already embedded within routine services despite being conducted amidst implementation challenges.

### Implication to practice

It is recommended that programmes supporting lower-level private-for-profit immunization service providers are encouraged to promote better data management practices. To build this capacity, barriers and coping mechanisms need to be analysed to identify weak points which can be strengthened to support data quality improvement efforts. There is a need to address challenges at all levels including data collection and management levels both at community, and facility as well as management levels such as sub-national and national health information system data management centres.

### Limitations

One limitation to this study was the self-report nature of the responses provided which informed the results that have been presented here. Therefore, while interpreting these results caution needs to be exercised to interpret them within the context in which data collection took place. Secondly, qualitative data collection took on a cross-sectional approach and we were only able to report results from data collected during the time of fieldwork. The strength of this study is that a diverse representation of private health service providers within an urban context participated in providing information as respondents. The multidisciplinary team of clinicians, health systems managers, researchers, public health professionals and data experts ensured verification of the information provided.

## Conclusion

Within the urban context, delivery of immunization services by lower-level private-for-profit service faces data quality challenges some of which stem from the characteristics of service users and the nature of urban health service delivery. However, key actors worked around these challenges to devise coping mechanisms and strategies to ensure that the data collected and submitted is of high quality. Key among the strategies were; Experiential attachment for practical skills acquisition in data management at public health facilities, Data quality peer-to-peer learning among private-for-profit immunization service providers, and registration using a cohort system for easy tracking of records during subsequent visits. Other coping strategies included; separating visitors’ records from regular attendants, service delivery modifications such as rewards for better performance, engagement of VHTs in outreaches in service provision and data completion, and data quality checks through review of monitoring charts to identify gaps in data quality. Improvement of data quality within this context as revealed by the study ought to optimally take advantage of policy provisions such as the ones mandating the public health facilities to supervise the private immunization service providers within their catchment areas. Nonetheless, there is also a need for interventions to respond to chronic barriers such as the high staff turnover among the private immunization service providers as long as finding solutions to long-term planning for the huge volume of non-resident service users who in most cases do not complete their immunization schedules from the city.

## Abbreviations

COREQ: Consolidated Criteria for Reporting Qualitative Results
DHIS: District Health Information System
DIT: Data Improvement Team
DMO: Division Medical Officer
DQA: Data Quality Assurance
EPI: Expanded Program on Immunization
HC: Health Centre
HMIS: Health Management Information Systems
HPV: Human Papilloma Vaccine
KCCA: Kampala Capital City Authority
NGO: Non-Governmental Organisation
PCV: Pneumococcal Conjugate Vaccine
PFP: Private-for-profit
PHC: Primary Health Care
PNFP: Private-not-for-profit
VHT: Village Health Teams
